# Reversible DNA i-motif to hairpin switching induced by copper(ii) cations†
†Electronic supplementary information (ESI) available: Experimental, preliminary experiments, data fitting and supporting data. See DOI: 10.1039/c5cc05111h



**DOI:** 10.1039/c5cc05111h

**Published:** 2015-08-07

**Authors:** Henry Albert Day, Elisé Patricia Wright, Colin John MacDonald, Andrew James Gates, Zoë Ann Ella Waller

**Affiliations:** a School of Pharmacy , University of East Anglia , Norwich Research Park , Norwich , NR4 7TJ , UK . Email: z.waller@uea.ac.uk; b School of Biological Sciences , University of East Anglia , Norwich Research Park , Norwich , NR4 7TJ , UK; c Centre for Molecular and Structural Biochemistry , University of East Anglia , Norwich Research Park , Norwich , NR4 7TJ , UK

## Abstract

i-Motif DNA structures have previously been utilised for many different nanotechnological applications, but all have used changes in pH to fold the DNA. Herein we describe how copper(ii) cations can alter the conformation of i-motif DNA into an alternative hairpin structure which is reversible by chelation with EDTA.

## Abstract 2

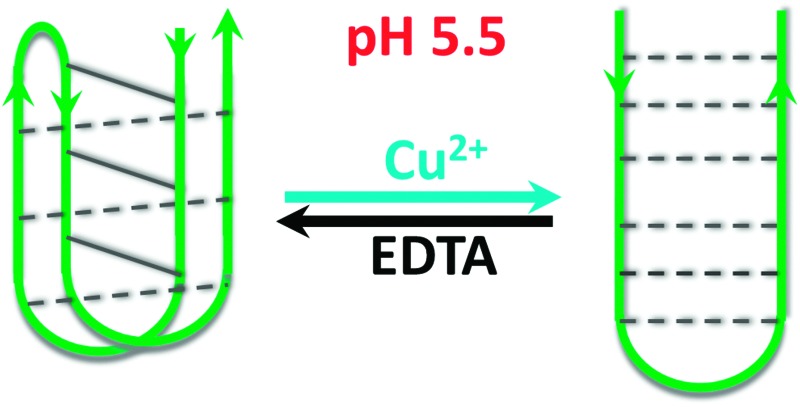
i-Motif forming DNA sequences have previously been used for many different nanotechnological applications, but all have used changes in pH to fold the DNA. Here it is shown that Cu(ii) cations can be used to re-fold i-motifs into hairpin structures, without changing the pH.

Research into the polymorphic nature of DNA has many applications, from elucidating the effects of forming such structures in biology, to the creation of nano-architectures and devices.^1^ DNA can form many different types of secondary structures, depending on the sequence of bases and are influenced by pH^2,3^ and the local environmental conditions such as the presence of cations^4,5^ or ligands.^6,7^ In contrast to Watson and Crick duplex, i-motifs are four-stranded nucleic acid secondary structures comprised of two parallel duplexes hydrogen bonded together in an antiparallel orientation by intercalated, cytosine^+^–cytosine base pairs.^8^ Such base pairing is driven by acidic conditions where the N3 in cytosine can protonate; consequential i-motif formation is rapid.^9,10^ Since 2003, this property has been exploited in the design of hundreds of pH-driven nanomachines^11^ such as the first proton-fuelled i-motif nanomotor,^2^ a device which can map spatial and temporal pH changes inside living cells^12^ and DNA “bipedal walkers”.^13^ The critical environmental changes for these i-motif-based DNA machines to function involve changing the pH to fold the structure. Following recent work indicating that Ag^+^ may fold i-motif DNA at neutral pH,^5^ we hypothesised that other cations could influence i-motif structure and function. The outcome of a preliminary screen indicated that Cu^2+^ could fold an i-motif forming DNA sequence from the human telomere (see ESI,† Section 2).

To investigate the type of structure which could be forming, UV difference spectroscopy was utilised to indicate the type of folded structure present after addition of Cu^2+^. DNA secondary structures absorb UV light differently when folded and unfolded and taking the difference between these spectra gives an indicative spectrum which can be used for characterisation.^14^ The thermal difference spectrum of human telomeric i-motif forming sequence at pH 5.5 (hTeloC, 5′-[TAA-CCC-TAA-CCC-TAA-CCC-TAA-CCC]-3′) gives a large positive change in absorbance at 235 nm and a negative change at 295 nm (Fig. 1). In the case of the addition of cations, we chose to perform the difference spectra at pH 7.4 and ambient temperature both before (unfolded) and after the addition of CuCl_2_ (folded). The resulting “Cu^2+^ difference” spectrum (Fig. 1) gives rise to a positive signal at 258 nm and a negative signal at 290 nm, both inconsistent with i-motif. As i-motif forming sequences also have potential to form hairpin structures,^15–17^ for comparison a known sequence capable of forming a hairpin, but not an i-motif structure in the presence of silver cations (hairpinC, 5′-[CTC-TCT-TCT-CTT-CAT-TTT-TCA-ACA-CAA-CAC-AC]-3′) was also used.^5,18^ The resulting “Ag^+^ difference” and “Cu^2+^ difference” spectra of the hairpinC sequence and the “Cu^2+^ difference” spectrum of hTeloC are almost identical, showing a positive signal at 258 nm, and a negative signal at 294 nm, consistent with a hairpin structure.^14^ Characterisation of other known i-motif forming sequences indicates this is observed across other types of C-rich sequences (see ESI,† Section 4.1). This finding supports the formation of a hairpin-type structure in i-motif forming sequences.

**Fig. 1:**
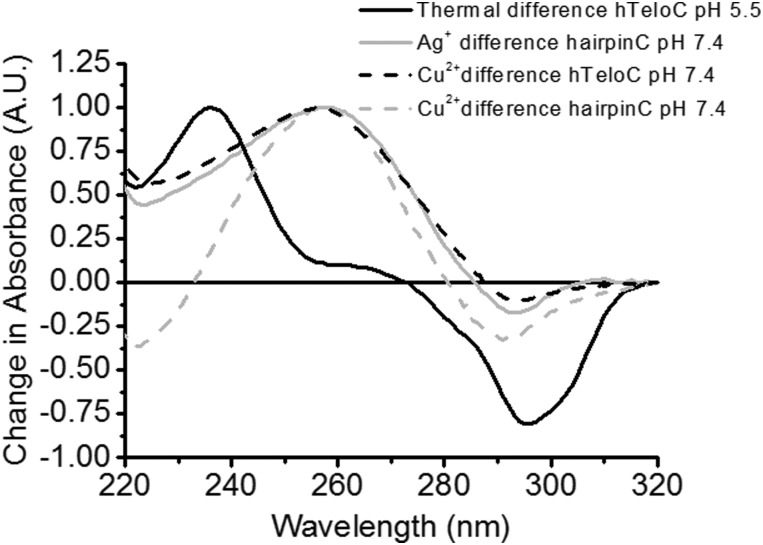
Comparison of UV thermal difference spectra of 2.5 μM hTeloC in 10 mM sodium cacodylate at pH 5.5 (black); copper-difference spectra at pH 7.4 of hTeloC (black dash) and hairpin (grey dash) at pH 7.4 using 250 μM of CuCl_2_ to form the folded conformations and silver-difference spectra at pH 7.4 of hairpinC using 15 μM of AgNO_3_ (grey).

To further investigate the changes observed, circular dichroism (CD) spectroscopy was used to study the effect of Cu^2+^ on the folding and structural conformation of hTeloC DNA. The CD spectrum of hTeloC in pH 5.5 buffer shows a negative signal at 255 nm and a positive signal at 288 nm, indicative of folded i-motif structure.^19^ However, the CD spectrum of hTeloC in pH 7.4 buffer shows a negative signal at 250 nm and a positive signal at 275 nm (Fig. 2a), indicative of a population which is mostly unfolded DNA.^19^ A titration of hTeloC with Cu^2+^ was performed where aliquots of CuCl_2_ were added to the DNA, mixed and a spectrum taken immediately. Upon titration of Cu^2+^ into a solution of hTeloC, there was a systematic bathochromic shift in the positive signal at 270 nm towards 280 nm. The positive peak at 220 nm and the negative peak at 248 nm decreased in intensity in response to addition of Cu^2+^.

**Fig. 2:**
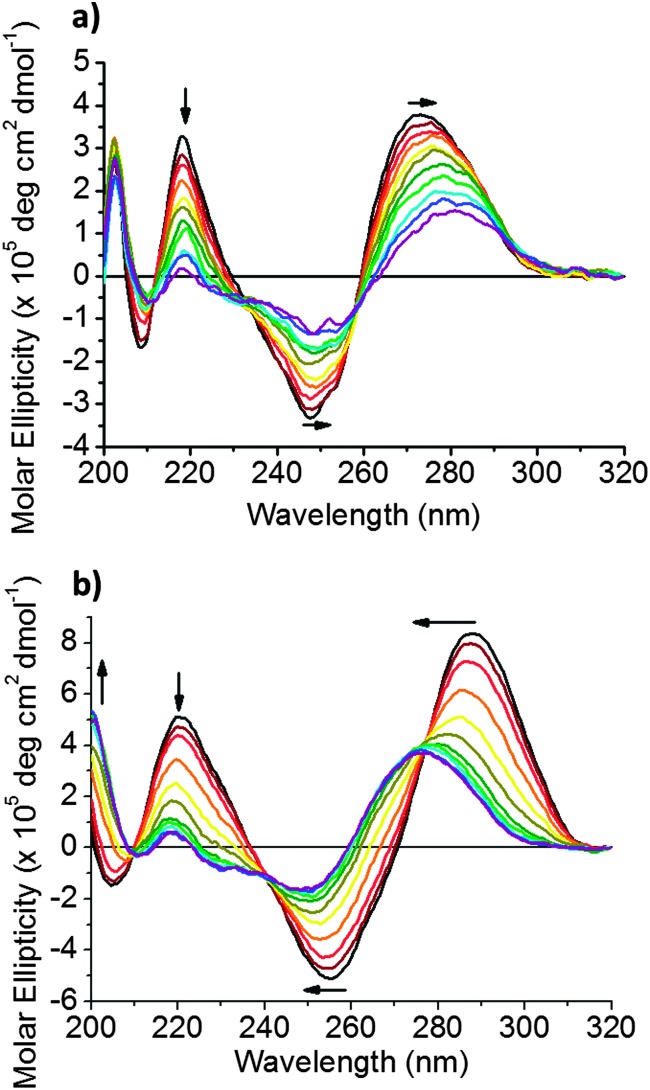
(a) CD spectra of 10 μM hTeloC in 50 mM sodium cacodylate buffer, pH 7.4 with 0–1 mM of CuCl_2_ added. (b) CD spectra of 10 μM hTeloC in 50 mM sodium cacodylate buffer, pH 5.5 with 0–1 mM of CuCl_2_ added.

This shift and the other spectral changes, framed by isoelliptic points at 215, 235 and 260 nm, indicates formation of a second population in solution which is not i-motif. However, the decrease in signal intensity and the quality of the spectra, particularly at the concentrations above 500 μM CuCl_2_, is affected by poor signal-to-noise, which may suggest some aggregation or precipitation of the DNA complex. To explore the effect of the cations in an environment where the sequence is predominantly in an i-motif conformation, the experiment was repeated in analogous buffer conditions at pH 5.5 (Fig. 2b). The initial population is folded into an i-motif with a characteristic positive peak at 288 nm and negative peak at 255 nm. On addition of up to 1 mM of Cu^2+^, the negative peak at 255 nm shifts to 250 nm. Similarly, the positive peak at 288 nm was also found to blue-shift on addition of Cu^2+^ to give rise to a broad peak at ∼278 nm; this was similar to the shape of the spectra after addition of Cu^2+^ at pH 7.4. Superficially, these changes appear to be consistent with the unfolding of i-motif DNA. However, the signals are not quite the same as those for unfolded DNA (see ESI,† Section 4.2).^19^ On closer inspection of the CD spectra of acid-stabilised i-motif in the absence and presence of Cu^2+^, when compared to unfolded DNA at pH 7.4 and the acid-stabilised hairpin forming sequence, reveals that there are further similarities between the C-hairpin and the acid stabilised i-motif in the presence of Cu^2+^ (see ESI,† Fig. S4): there is a peak at 200 nm, which is present in each of these samples, which is almost absent in the acid stabilised i-motif sample and red-shifted in the single-stranded i-motif forming sequence at pH 7.4. This indicates an additional peak to suggest the formation of this alternative structure. The formation of an alternative structure, rather than unfolding is further supported by the changes in structure observed at pH 7.4, where the majority of DNA is already unfolded before addition of Cu^2+^, yet a second conformation is still observed to form when Cu^2+^ is added.

In contrast to the CD experiments performed at pH 7.4, none of the spectra at pH 5.5 show poor signal to noise, or any other signs of precipitation. To give a measurement of the changes observed, the ellipticity at 288 nm was plotted against the concentration of CuCl_2_ added (Fig. 3). The sigmoidal shape of the binding curve indicates a cooperative effect; we fitted this to the Hill 1 equation using Origin (see ESI,† Section 3) and obtained a Hill coefficient (*n*) of 2.5 (±0.3), indicating positive cooperativity (*n* > 1). Additionally, the half-copper(ii) cation concentration for the transition ([Cu^2+^]_50_) between the two states was found to be 382 (±14) μM. The high concentration compared to DNA (10 μM) would suggest that the Cu^2+^ interacts by stabilising the sugar-phosphate backbone in the hairpin species rather than mediating specific base pairing as observed with Ag^+^.^5^ It has previously been proposed that an alternative hairpin conformation exists in equilibrium with acid-stabilised i-motif,^16^ addition of Cu^2+^ could shift this equilibrium towards the hairpin structure. i-Motif structure has two wide grooves and two narrow grooves^20^ whereas a hairpin will have grooves similar in size to that of B-DNA. The stability of i-motif structures is closely related to the interactions between phosphodiester backbones in the narrow grooves and sugar–sugar contacts are important to offset repulsion from the phosphates as the strands become close together.^21^ The coordination of cations along the backbone could affect these interactions in both a positive and negative way. It is possible that Cu^2+^ supports stabilisation of the sugar phosphate backbones as they come together in the grooves in the hairpin but not the i-motif.

**Fig. 3:**
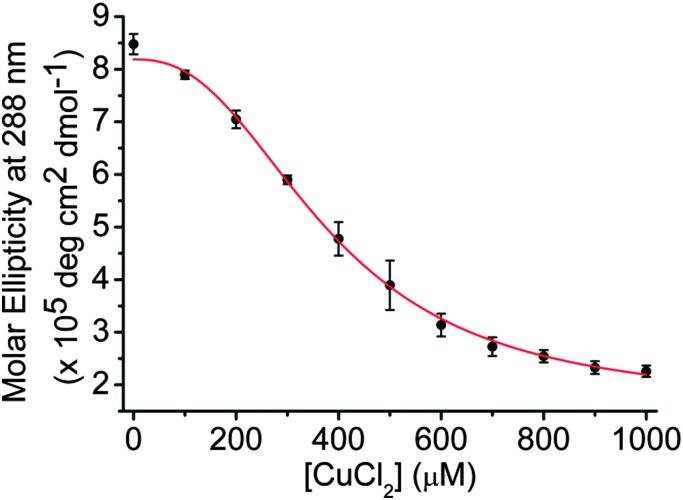
Plot of change in molar ellipticity at 288 nm on addition of between 0 and 1 mM of CuCl_2_ to 10 μM of hTeloC in 50 mM sodium cacodylate, pH 5.5. Error bars show standard deviations across three repeats.

Due to the rapid changes which occur directly after mixing (within the timescale of the CD scan), this suggested the alternative structure formed could be intramolecular, as intermolecular formation can take much longer.^22^ UV thermal melting experiments at different concentrations of DNA showed little variability in the melting temperature of the DNA structure, consistent with a predominantly intramolecular process^23,24^ (see ESI,† Section 4.3). Furthermore, initial kinetics experiments monitoring the CD at 288 nm after addition of Cu^2+^ gave rise to a characteristic folding time of 44 ± 2 s (see ESI,† Section 4.4). This indicates folding occurs on a similar timescale to i-motif formation when changing the pH from 8 to 6, but not as fast as formation at pH 5.^9^

We were interested in examining the reversibility of this folding-type process. EDTA is a classical chelating agent and can chelate many different types of cations^25^ including Cu^2+^. As such, we analysed the effect of adding EDTA in after titration with Cu^2+^ using CD spectroscopy. At pH 5.5, the CD spectrum resembles a predominantly i-motif DNA population, after addition of 1 mM (100 eq.) of Cu^2+^, the CD spectrum changes to the alternative conformation. After adding EDTA to this solution, a return to a spectrum consistent with that of an acid stabilised i-motif is observed once more, *i.e.* it returns to the form it previously took before addition of Cu^2+^ (Fig. 4a). As this type of conformational change could have applications in nanotechnology, we were interested to determine whether several cycles of conformational change were possible. Sequential additions of Cu^2+^ and EDTA showed that multiple cycling is possible (Fig. 4b and ESI,† Fig. S8). These results showed that the formation of the Cu^2+^-structure is reversible on addition of EDTA. It was found that to return the structure to the previous form, a slight excess of EDTA was required after addition of Cu^2+^ (see ESI,† Fig. S7). Nevertheless, multiple cycling is possible, even when equal proportions of each agent are added.

**Fig. 4:**
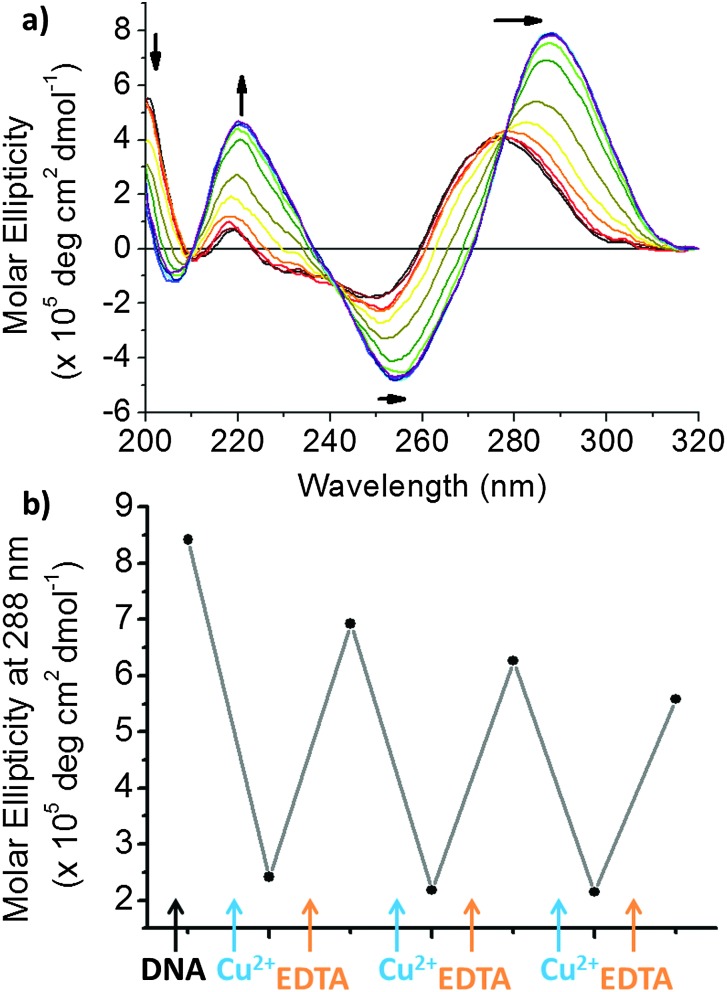
(a) CD spectra of 10 μM hTeloC in 50 mM sodium cacodylate buffer, pH 5.5 with 1 mM of CuCl_2_ and 0–1 mM of EDTA added. (b) CD spectra of 10 μM hTeloC in 50 mM sodium cacodylate buffer, pH 5.5 with 1 mM of CuCl_2_ and 0–1 mM of EDTA added.

To further characterise what type of structure the DNA adopts on addition of Cu^2+^ we decided to use one dimensional ^1^H NMR. Previous work by Hurley and co-workers used the imino proton signals to monitor transition from i-motif to hairpin on addition of small molecule ligands.^17^ To replicate the conditions used in the CD we performed the experiments under the same buffer conditions and DNA concentration. In the absence of Cu^2+^, imino proton peaks are observed at 15.5 ppm, which are characteristic of the C≡C^+^ base pairs in an i-motif (Fig. 5). After addition of 1 mM of Cu^2+^, additional signals at 12.5 ppm also appear. These new signals are consistent with formation of a hairpin-type structure and are present in the same region observed by Hurley and co-workers in their ligand work.^4,17^ As Cu^2+^ is paramagnetic, broadening in the overall signals was also observed. Nevertheless, imino-proton signals for C≡C^+^ base pairs are still detected on addition of the Cu^2+^, indicating that the hairpin is composed of both C≡C^+^ and Watson–Crick base pairs. To reverse the effect, 1 mM EDTA was added. After mixing, the peaks at 12.5 ppm (hairpin) disappeared, indicating the return towards i-motif conformation. Taken together with the UV difference and CD spectroscopy, this indicates addition of Cu^2+^ to acid-stabilised i-motif alters the structure to a hairpin conformation.

**Fig. 5:**
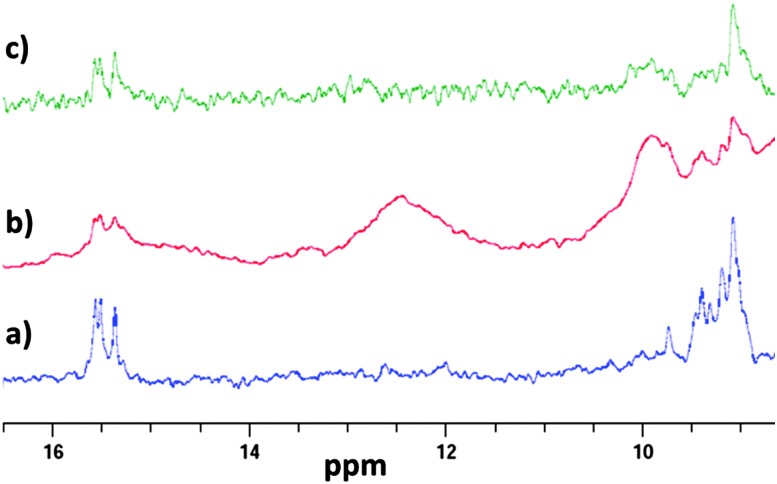
1D ^1^H NMR of 10 μM hTeloC in 50 mM sodium cacodylate buffer, pH 5.5 with 5% D_2_O (a); with 1 mM of CuCl_2_ (b); with 1 mM of CuCl_2_ and 1 mM of EDTA (c).

This research indicates that Cu^2+^ can be used to alter the conformation i-motif DNA structure at room temperature and acidic pH into a hairpin-type structure. This process is reversible with the addition of EDTA and does not require any thermal annealing. This offers an alternative conformational switch using different conditions, without changing the pH. Like previous studies which have constructed DNA logic gates utilising a H^+^/Ag^+^ induced i-motif structure;^26^ this work could be exploited in analogous i-motif DNA-based nanomachines, logic gates or sensors as there are potentially three structural outputs (single strand, i-motif and hairpin) which can be achieved with the same oligonucleotide sequence through changing pH or cationic conditions.

We thank Dr Myles Cheesman of the Henry Wellcome Laboratories for Biological Chemistry, UEA, for the use of the CD spectrometer and Novartis for a studentship (HAD). This work was supported by a Royal Society grant (RG140746) and EPW is supported by a BBSRC grant (BB/L02229X/1).
