# Supplementary material for: Reversible DNA i-motif to hairpin switching induced by copper(ii) cations
Source: Chem Commun (Camb). 2015 Aug 7;51(74):14099–102. doi: 10.1039/c5cc05111h (PMC4563791; doi:10.1039/c5cc05111h)
Supplement: Supplementary file 1 [file CC-051-C5CC05111H-s001.pdf]

Electronic Supplementary Material (ESI) for ChemComm.
This journal is © The Royal Society of Chemistry 2015

Reversible DNA i-Motif to Hairpin Switching Induced by Copper (II) Cations.

Henry A. Day,[a] Elise P. Wright,[a] Colin J. MacDonald,[a] Andrew J. Gates,[b,c] and Zoë A. E. Waller[a,c]*

[a]School of Pharmacy, University of East Anglia, Norwich Research Park, Norwich, NR4 7TJ, UK

[b]School of Biological Sciences, University of East Anglia, Norwich Research Park, Norwich, NR4 7TJ, UK

[c]Centre for Molecular and Structural Biochemistry, University of East Anglia, Norwich Research Park, Norwich, NR4 7TJ, UK

SUPPORTING INFORMATION for *Chem. Commun.*

Contents

1. GENERAL EXPERIMENTAL……S2
   1.1 FRET MELTING……S2
   1.2 CIRCULAR DICHROISM EXPERIMENTS……S3
   1.3 UV EXPERIMENTS……S3
   1.4 ^{1}H NMR EXPERIMENTS……S4
2. PRELIMINARY EXPERIMENTS……S5
3. DATA FITTING……S8
4. ADDITIONAL SUPPORTING DATA……S8
   4.1 “COPPER DIFFERENCE” SPECTRA……S8
   4.2 COMPARISON OF CD SPECTRA……S9
   4.3 INTRA VS INTERMOLECULAR STRUCTURE FORMATION……S10
   4.4 FOLDING KINETICS……S12
   4.5 REVERSIBILITY WITH EDTA……S15
5. SUPPORTING INFORMATION REFERENCES……S17

1. GENERAL EXPERIMENTAL

All the oligonucleotides (ODNs) and their fluorescent conjugates were purchased from Eurogentec and were HPLC purified. Solid DNA samples were initially dissolved as a stock solution in MilliQ water (100 μM for labelled and 1 mM for un-labelled ODNs); further dilutions were carried out in the respective sodium cacodylate buffer. Annealed samples were thermally annealed in a heat block at 95°C for 5 minutes and cooled slowly to room temperature overnight. Non-annealed samples had the DNA diluted into the respective buffer and were used immediately.

1.1 FRET MELTING EXPERIMENTS

The ability of cations to affect the stability of i-motif DNA was assessed using a fluorescence resonance energy transfer (FRET) DNA melting based assay. The labelled oligonucleotide $hTeloC_{FRET}$ (5'-FAM-d[TAA-CCC-TAA-CCC-TAA-CCC-TAA-CCC]-TAMRA-3'; donor fluorophore FAM is 6-carboxyfluorescein; acceptor fluorophore TAMRA is 6-carboxytetramethyl-rhodamine) was prepared as a 400 nM solution in buffer containing 10 mM sodium cacodylate (pH 7.4) with 5 mM NaCl and then thermally annealed. $CuCl_2$ was dissolved in purified water. Strip-tubes (QIAgen) were prepared by aliquoting 10 μL of the annealed DNA, followed by 10 μL of the cation solutions. Fluorescence melting curves were determined in a QIAgen Rotor-Gene Q-series PCR machine, using a total reaction volume of 20 μL. Measurements were made with excitation at 483 nm and detection at 533 nm. Final analysis of the data was carried out using QIAgen Rotor-Gene Q-series software and Origin.

1.2 CIRCULAR DICHROISM EXPERIMENTS

Circular dichroism (CD) spectra were recorded on a Jasco J-810 spectropolarimeter using a 1 mm path length quartz cuvette. Human telomeric i-motif (hTeloC, 5'-d[TAA-CCC-TAA-CCC-TAA-CCC-TAA-CCC]-3') was diluted in a buffer containing sodium cacodylate (10 mM or 50 mM and pH 7.4 or 5.5 as detailed) to achieve a total volume of 200 µL. The scans were performed at 20°C over a wavelength range of 200-320 nm with a scanning speed of 200 nm/min, a response time of 1 s, 0.5 nm pitch and 2 nm bandwidth. A blank sample containing only buffer was treated in the same manner and subtracted from the collected data. Solutions of $CuCl_2$ and EDTA were added in small aliquots to the desired equivalent proportions using a pipette. The CD spectra represent an average of three scans and are zero corrected at 320 nm. Kinetics experiments were performed using the time course management function in the Jasco software using single measurements every 0.5 s at 288 nm. Each experiment was performed in triplicate. Final analysis and processing of the data was carried out using Origin.

1.3 UV EXPERIMENTS

UV spectroscopy experiments were performed on a Agilent Technologies Cary 60 UV-Vis spectrometer equipped with a Quantum Northwest TC1 thermal peltier controller and recorded using a low volume quartz cuvette. The thermal melting curves were obtained by monitoring the absorbance at 295 nm. Samples (200 µL) were prepared then transferred to a masked quartz cuvette (1 cm path length), covered with a layer of silicone oil and stoppered to reduce evaporation of the sample. Samples were held at 4°C for 5 minutes then heated to 95°C three times at a rate of 0.5°C/min, each with a 5 minute hold at 4°C and

95°C and data was recorded every 1°C during both melting and annealing. Each point was the average of three scans. Melting temperatures (T_m) were determined using the first derivative method.

For UV difference spectra, human telomeric i-motif (hTeloC, 5'-d[TAA-CCC-TAA-CCC-TAA-CCC-TAA-CCC]-3'), i-motif forming sequences from the c-Myc promoter (5'-d[TCC-CCA-CCT-TCC-CCA-CCC-TCC-CCA-CCC-TCC-CCA]-3'),[1] hif-1-α promoter (5'-d[CGC-GCT-CCC-GCC-CCC-TCT-CCC-CTC-CCC-GCG-C]-3') and PDGF-A (5'-d[CCG-CGC-CCC-TCC-CCC-GCC-CCC-GCC-CCC-GCC-CCC-CCC-CCC-CC]-3')[2] or the control sequence capable of forming a hairpin (hairpinC, 5'-[CTC-TCT-TCT-CTT-CAT-TTT-TCA-ACA-CAA-CAC-AC]-3')[3] were diluted in a buffer containing sodium cacodylate (10 mM, pH 7.4) to achieve a concentration of 2.5 µM and a total volume of 200 µL. The DNA was not annealed and used immediately after dilution. A solution of $CuCl_2$ (or $AgNO_3$ for the comparison) was added in small aliquots until the desired amount using a Hamilton syringe. Spectra were recorded over a wavelength range of 400 - 200 nm at 20°C in the absence of any additional cations and then in the presence of up to 100 eq of $CuCl_2$ (or 6 eq of $AgNO_3$) and zero corrected at 400 nm. Spectra of the buffer and $CuCl_2$ (or $AgNO_3$) were recorded and subtracted from the spectra of the DNA. The difference spectrum was calculated by subtraction of the folded (in the presence of $CuCl_2$) spectrum from the unfolded spectrum (in the absence of $CuCl_2$) and normalised so the maximum change in absorption was set to +1 as previously described.[4]

1.4 ^{1}H NMR EXPERIMENTS

^{1}H NMR experiments were performed using a Bruker Avance III 800 MHz spectrometer equipped with an HCN inverse triple resonance z-gradient probe. Aqueous solutions were

prepared with the addition of 5% D_2O to enable field/frequency lock. Solvent suppression of the water resonance was achieved using a 1D Watergate sequence employing a symmetrical 3–τ–9–τ–19 pulse train inversion element. The solvent resonance, which was minimized, was set on-resonance at the transmitter offset and the interpulse delay time (τ) was adjusted to achieve an excitation maximum in the imino proton region of interest. The hTeloC oligonucleotide sequence was diluted to a concentration of 10 µM in pH 5.5 50 mM sodium cacodylate buffer containing 5% D_2O. The spectrum of hTeloC alone was measured over 1 hour after which 1 mM of $CuCl_2$ was added and the subsequent spectrum acquired over 2 hours. Finally 1 mM EDTA was added and the spectrum acquired again for 1 hour. NMR spectra were acquired and processed using Bruker's TopSpin™ software package (v3.1.7 Bruker Biospin) for NMR data analysis.

2. PRELIMINARY EXPERIMENTS

The FRET melting experiments monitor the emission of the donor fluorophore (FAM) as the temperature of the sample is increased. When the DNA is folded, the fluorophores are close together and FAM is quenched; as the temperature increases, the DNA melts, FAM and TAMRA move further apart and FAM is no longer quenched. This results in an increase in the fluorescence of the donor fluorophore FAM; we can use these signals to indicate how much of the sample is folded and also monitor DNA melting.

The initial experiments screened between 10 µM and 100 mM concentrations of cations, added to $hTeloC_{FRET}$ in 10 mM pH 7.4 buffer. A selection of salts were screened, with cations varying in size, charge and geometries: $AlCl_3$, $CdCl_2$, $CoCl_2$, $CuCl_2$, $Ga(NO_3)_3$, $HoCl_3$, $In(NO_3)_3$,

$FeCl_3$, $PbAc_2$, $MnCl_2$, $HgAc_2$, $NiCl_2$, $RuCl_3$, $SmCl_3$, $SrCl_2$, TlAc, $TbCl_3$, $YbCl_3$, YCl_3, $ZnAc_2$. After the initial screen, several hits were identified which seemed to cause folding at neutral pH at 10 or 100 µM: $AlCl_3$, $CuCl_2$, $Ga(NO_3)_3$, $HoCl_3$, $SmCl_3$, $TbCl_3$, $YbCl_3$, YCl_3, $HgAc_2$ and $ZnAc_2$ (Fig. S1). At pH 7.4 the majority of $hTeloC_{FRET}$ will be unfolded at ambient temperature and give a high level of fluorescence. As the DNA is normally unfolded at pH 7.4, it is not possible to measure a melting temperature in the absence of the cations however, these cations have induced significantly higher DNA melting temperatures, suggesting they form stable secondary structures.

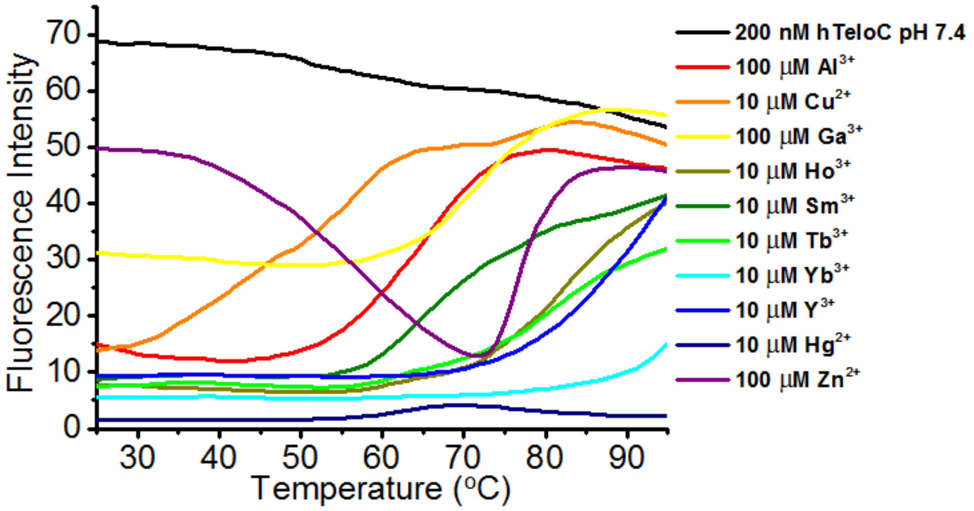

Figure S1: Representative FRET melting experiments of 0.2 µM $hTeloC_{FRET}$ in 10 mM sodium cacodylate buffer, pH 7.4 supplemented with 5 mM of NaCl and different cations as specified

To observe the type of secondary structure forming, the effect of the "hit" cations on the i-motif forming DNA sequence was initially assessed by CD. Due to the higher concentration of DNA used in CD compared to the FRET screen (10 µM compared to 200 nM), testing the cations at the same concentrations as the FRET based screen (80 – 100 µM) resulted in very little change in the CD spectra. Experiments at the same number of equivalents required 4-5

mM cations; at these concentrations, precipitation was apparent in the presence of Ho^{3+}, Sm^{3+}, Tb^{3+} and Yb^{3+}. Moreover, after addition of Al^{3+}, Ga^{3+} and Zn^{2+}, although changes in the spectra were observed by CD; the concentrations of cations required were outside the limit of the buffering capabilities of the buffer and the changes observed were likely to be due to changes in pH. Nevertheless, adding 80 µM of $CuCl_2$ to hTeloC at pH 7.4 resulted in a change in the CD spectrum (Fig. S2); the positive peak at 274 nm shows a bathochromic shift to 278 nm and an increase in ellipticity whereas the negative peak at 248 nm also shows a similar effect, shifting to 250 nm. These changes are consistent with a change in the conformation of the DNA, from random coil to a folded DNA structure. The bands are not consistent with formation of i-motif and are more similar to what would be expected from double-helical DNA. Over 80 µM of $CuCl_2$ started to affect the buffering capability of the sodium cacodylate.

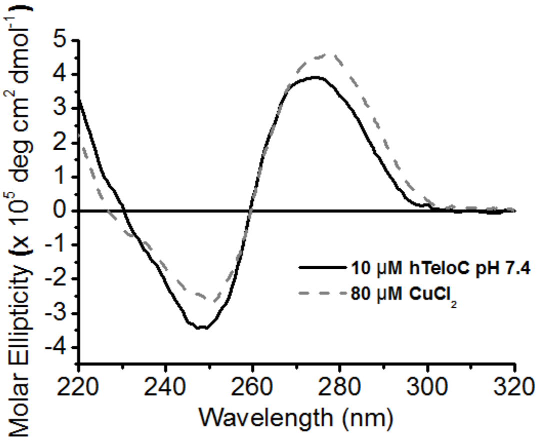

Figure S2: CD spectra of 10 µM hTeloC in 10 mM sodium cacodylate buffer, pH 7.4 supplemented with 5 mM of NaCl (black) and 80 µM of $CuCl_2$ (grey dashes).

For the remaining experiments, 50 mM sodium cacodylate was used to enable use of higher concentrations of $CuCl_2$. This has two effects though, the buffering capacity increased, but

also the concentration of sodium cations, which are known to have a destabilizing effect on i-motif structure.[5] Our initial screen indicated that $CuCl_2$ was able to fold the DNA at pH 7.4 and the DNA was found to melt at 57°C. Given the DNA is completely unfolded at physiological pH at the start of the experiment (25°C) this indicates a ΔT_m of at least +32°C on addition of 10 µM $CuCl_2$ to 200 nM hTeloC$_{FRET}$.

3. DATA FITTING

The sigmoidal curves were fitted to the Hill 1 equation using Origin using the standard constraints at the start and end values:

$$[\theta] = \frac{[Cu^{2+}]^n}{{K_A}^n + [Cu^{2+}]^n}$$ **Equation S1.**

Where $[\theta]$ is the molar ellipticity at 288 nm, K_A is the apparent association constant of binding Cu^{2+} and n is the Hill coefficient.

4. ADDITIONAL SUPPORTING DATA

4.1. "COPPER DIFFERENCE" SPECTRA

To investigate how different i-motif forming sequences appear in the "copper difference" spectra sequences from c-Myc, hif-1-α and PDGF-A were also examined in addition to the human telomeric sequence and hairpin control (Fig. S5). Each has a similar shape, consistent with formation of a hairpin-type structure, not an i-motif.

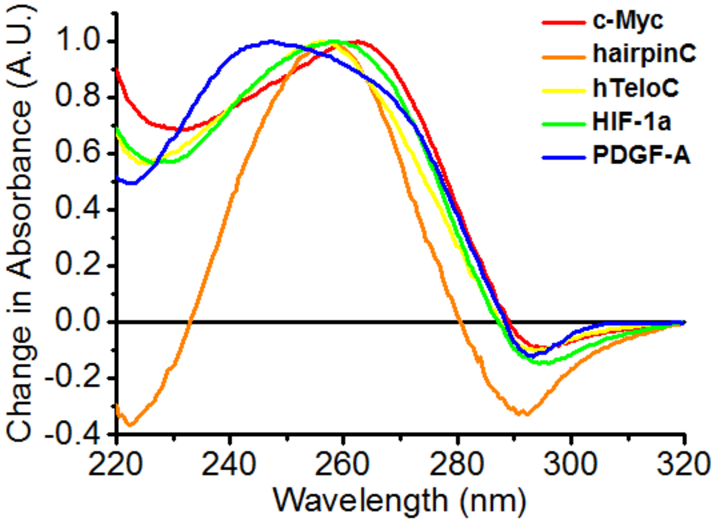

Figure S3: Comparative "Copper difference" spectra of 2.5 μM DNA in 50 mM sodium cacodylate buffer at pH 5.5 with 250 μM $CuCl_2$ with i-motif forming sequences from c-Myc, hTelo, hif-1-α and PDGF-A and a hairpin forming sequence

4.2 COMPARISON OF CD SPECTRA

To compare the differences between, acid stabilised i-motif, single stranded i-motif forming sequence and copper stabilised structures, the CD of each are stacked on top of each other in Fig S6. DNA in a random coil has a positive signal at 275 nm, whereas the spectra observed for hTeloC in the presence of 100 eq of $CuCl_2$ and hairpinC at pH 5.5 have a positive signal closer to 278-280 nm, which is not quite far enough for i-motif formation (typically 288 nm). Moreover the positive signals at 200 nm indicate hairpin formation, in Fig S4 is it possible to see how similar the copper-hTeloC complex and hairpinC appear in this region. Moreover, in each case the positive signal at 220 nm is also reduced. These subtle differences indicate that in the presence of copper cations, hTeloC is in a folded structure which is not i-motif.

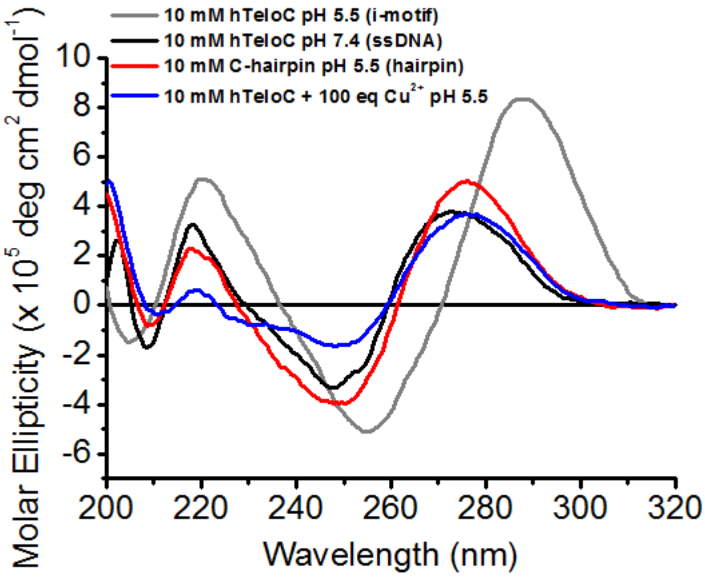

Figure S4: Comparative CD spectra of 10 µM DNA in 50 mM sodium cacodylate buffer: hTeloC at pH 5.5 (grey, i-motif), pH 7.4 (black, single stranded DNA) and pH 5.5 with 1 mM $CuCl_2$; hairpinC at pH 5.5 (hairpin).

4.3 INTRA VS INTERMOLECULAR STRUCTURE FORMATION

The nature of the fast conformational changes indicate formation of an intramolecular structure.[6, 7] However, to give an indication of the stability, folding and kinetics of the Cu^{2+} complex, UV thermal melting annealing experiments were also performed. The change in absorbance at 295 nm was measured as a function of temperature using different concentrations of DNA (Fig. S5). The absorbance vs temperature profiles for the heating and cooling transitions were found to be non-superimposable both in the absence (Fig. S5) and presence (Fig S6) of Cu^{2+}. The hysteresis show that the thermally-induced transitions follow slow kinetics under the experimental conditions. However, they do not change with concentration of DNA (Table S1), indicative of an intramolecular process.

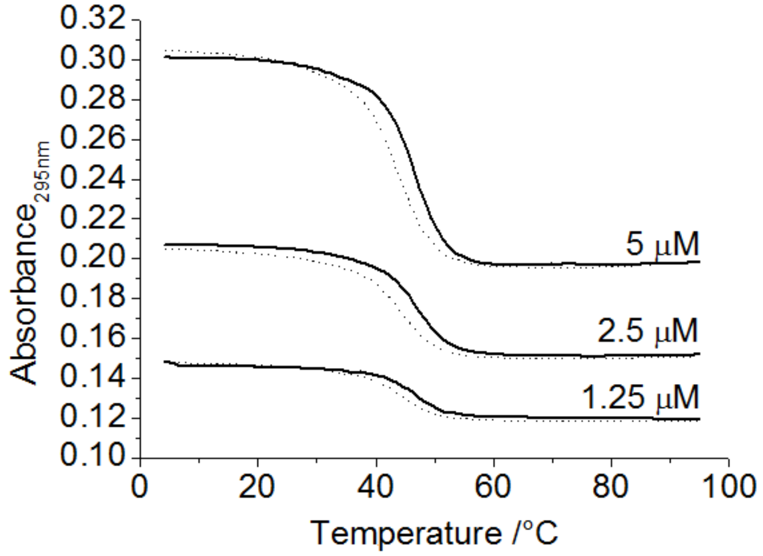

Figure S5: Example thermal melting (solid) and annealing (dotted) profiles in 50 mM sodium cacodylate buffer at pH 5.5 with different concentrations of DNA as detailed.

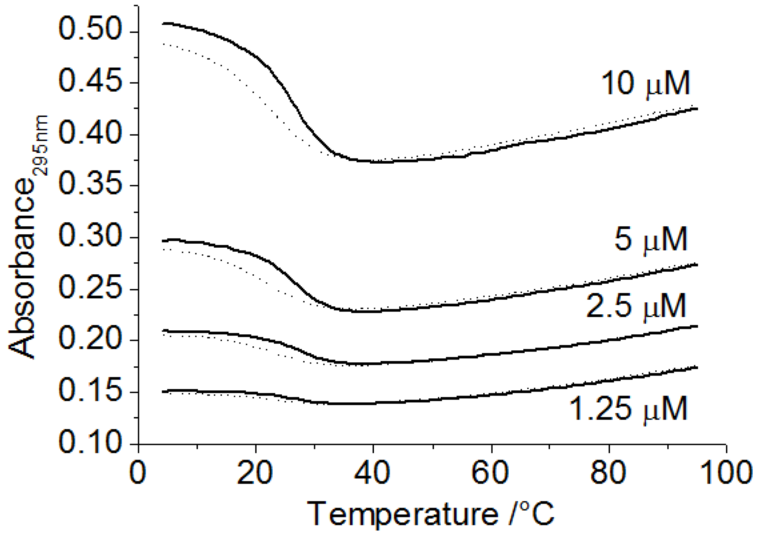

Figure S6: Example thermal melting (solid) and annealing (dotted) profiles in 50 mM sodium cacodylate buffer at pH 5.5 with 250 µM $CuCl_2$ and different concentrations of DNA as detailed.

Table S1: Melting and annealing temperatures of hTeloC in 50 mM sodium cacodylate buffer at pH 5.5 with different concentrations of $CuCl_2$ and DNA as detailed.

[hTeloC] /µM	[$CuCl_2$] /µM	$T_{melting}$ /°C	$T_{annealing}$ /°C	$T_{hysterisis}$ /°C
1.25	0	46	44	2
2.5	0	46	46	0
5	0	46	44	2
1.25	250	25	21	4
2.5	250	27	23	4
5	250	26	22	4
10	250	25	22	3

4.4 FOLDING KINETICS

To give an indication of the timescale it takes for the structure to turn from i-motif to hairpin, kinetics experiments were performed by mixing DNA and $CuCl_2$ and monitoring loss of signal at 288 nm in the CD spectra over time. As 10 µM of hTeloC at pH 5.5 showed complete conversion to the alternative structure at 1 mM $CuCl_2$, the experiments were performed in triplicate under conditions analogous to the previous experiments (see Fig. S9). As the concentration of Cu^{2+} >> DNA, we considered the conditions as pseudo-first order and the folding data was fitted to a single exponential function as previously described:[8]

$$\theta = A\,exp^{-t/t_1} + \theta_0 \qquad \textbf{Equation S2.}$$

Where θ is the ellipticity at 288 nm, t_1 is the characteristic folding time and gives $1/t_1$ as the characteristic folding rate.

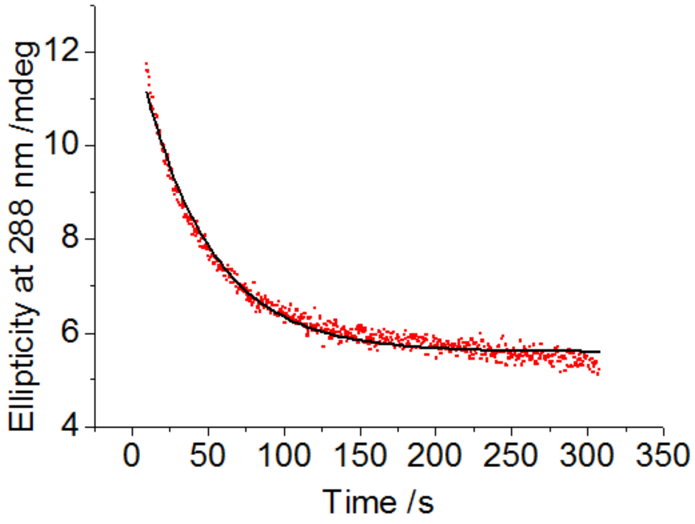

Figure S9: Example data monitoring the loss of i-motif signal at 288 nm in the CD using 10 μM hTeloC in 50 mM sodium cacodylate buffer, pH 5.5 with 1 mM of $CuCl_2$.

To give an indication of the reverse folding with EDTA, we also investigated the kinetics after addition of folding to hairpin, back to i-motif using EDTA (Fig. S10).

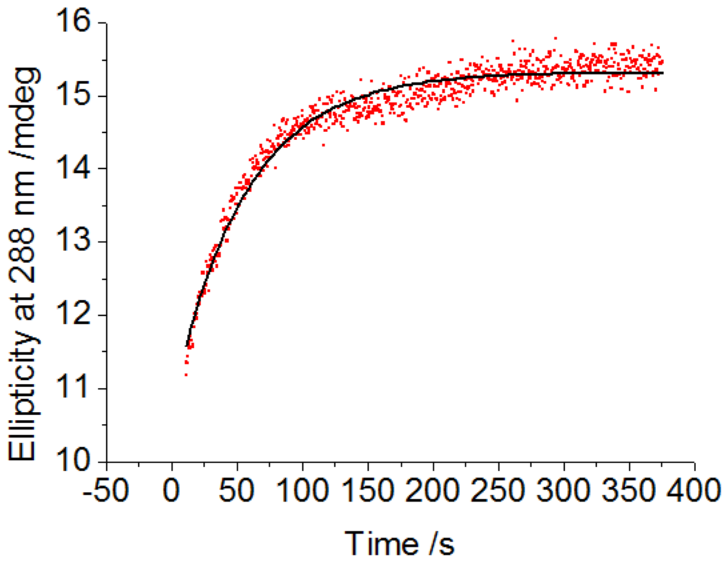

Figure S10: Example data monitoring the gain of i-motif signal at 288 nm in the CD using 10 μM hTeloC in 50 mM sodium cacodylate buffer, pH 5.5 with 1 mM of $CuCl_2$ and then 1 mM of EDTA added.

The rates of folding for different concentrations of $CuCl_2$ were investigated. A summary of the characteristic folding times and rates are given in Table S2.

Table S2: Average characteristic folding times and rates of hTeloC in 50 mM sodium cacodylate buffer at pH 5.5 with different concentrations of $CuCl_2$ and EDTA as detailed. Errors are the standard deviation from three repeats. *Indicates folding from hairpin to i-motif.

[hTeloC] /μM	[$CuCl_2$] /mM	[EDTA] /mM	t_1 /s	k /s^{-1}
10	0.5	0	104.99 ± 15.7	0.0097 ± 0.0015
10	0.75	0	63.16 ± 4.88	0.0159 ± 0.0012
10	1	0	42.58 ± 1.71	0.0235 ± 0.0009
10	1	1	56.66 ± 13.6*	0.0187 ± 0.0044*
10	1.25	0	46.21 ± 7.52	0.0221 ± 0.0038
10	1.5	0	42.22 ± 8.80	0.0245 ± 0.0058

From the characteristic folding times and rates, it is possible to observe that the rate reaches a plateau when the folding process is independent of Cu^{2+} concentration (i.e. [Cu^{2+}] >> the [Cu^{2+}]$_{50}$, calculated from the titration in Fig. 2b). At this point an estimate of the upper limit of the characteristic folding time (t_1) can be given as 44 s. The values obtained for the reverse-folding using EDTA were approximately the same within error.

Although these experiments give an insight into how quickly the folding process occurs, the drawback is the dead time of the instrument from addition of the Cu^{2+} or EDTA and the first measurements, typically about 8 s. This means that the measurement of the initial rate is not possible. It is also likely that the folding and re-folding processes go *via* at least one

intermediate, but without this initial data, it is not possible to make any firm analysis. To gain full insights into the full kinetics underlying these processes is beyond the scope of this present study; further experiments using stopped-flow would be required.

4.5 REVERSIBILITY WITH EDTA

To complement the fitting in Fig 4., we also performed the same analysis with the addition of EDTA to reverse the folding process (Fig. 5). Plotting the molar ellipticity at 288 nm against concentration of EDTA also gave a sigmoidal shaped curve (Fig. S7), fitting this with the Hill1 equation (Eq. S1) gave a Hill coefficient (*n*) of 5.4 (± 0.7), indicating positive cooperativity (n > 1). Additionally, the half-EDTA concentration for the transition ($[EDTA]_{50}$) between the two states was found to be 531 (± 24) µM, indicating that an excess of EDTA is required to reverse the folding completely (compared to 382 (± 14) µM for $CuCl_2$). Multiple cycling between each folded structure is possible with sequential additions of $CuCl_2$ and EDTA (Fig. 6 and S8), due to the excess EDTA required to return the folded structure to i-motif, when sequential aliquots of equimolar amounts of $CuCl_2$ and EDTA were added, a slight decrease in signal at 288 nm (i-motif) is observed with each cycle.

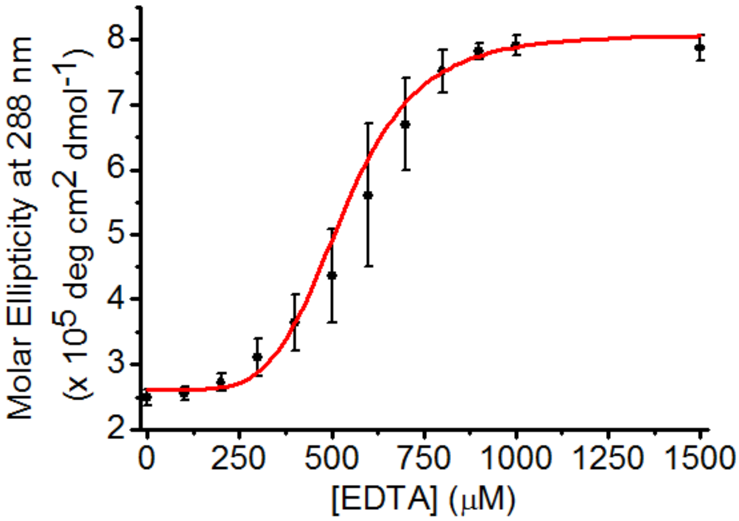

Figure S7: Plot of change in molar ellipticity at 288 nm on addition of between 0 and 1500 µM of EDTA to 10 µM of hTeloC in 50 mM sodium cacodylate, pH 5.5 with 1 mM $CuCl_2$. Error bars show standard deviations across three repeats.

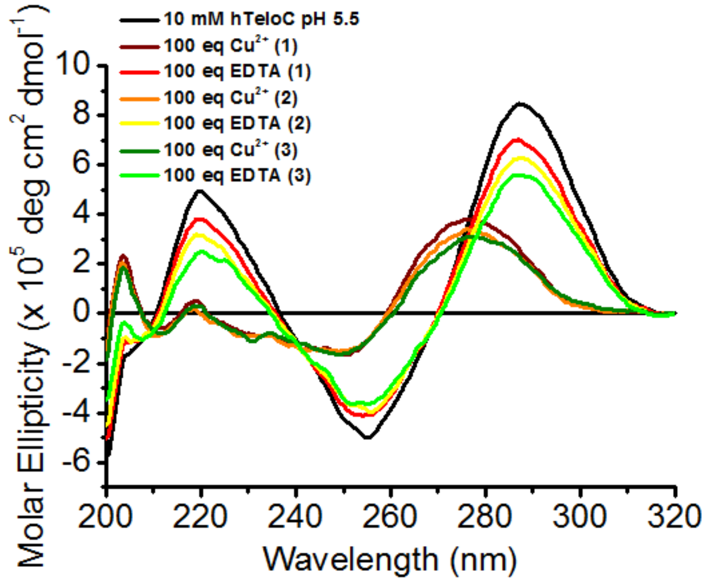

Figure S8: CD spectra of 10 µM hTeloC in 50 mM sodium cacodylate buffer, pH 5.5 with sequential additions of 1 mM of $CuCl_2$ and 1 mM of EDTA as detailed.

5. SUPPORTING INFORMATION REFERENCES

1. D. Sun and L. H. Hurley, *J. Med. Chem.*, 2009, **52**, 2863-2874.
2. J. A. Brazier, A. Shah and G. D. Brown, *Chem. Commun.* 2012, **48**, 10739-10741.
3. A. Ono, S. Cao, H. Togashi, M. Tashiro, T. Fujimoto, T. Machinami, S. Oda, Y. Miyake, I. Okamoto and Y. Tanaka, *Chem. Commun.* 2008, 4825-4827.
4. J. L. Mergny, J. Li, L. Lacroix, S. Amrane and J. B. Chaires, *Nucleic Acids Res.*, 2005, **33**, e138.
5. J.-L. Mergny, L. Lacroix, X. Han, J.-L. Leroy and C. Helene, *J. Am. Chem. Soc.*, 1995, **117**, 8887–8898.
6. E. E. Merkina and K. R. Fox, *Biophys. J.*, 2005, **89**, 365-373.
7. V. V. Jolad, F. K. Murad, J. R. Arnold and J. Fisher, *Org. Biomol. Chem.*, 2005, **3**, 2234-2236.
8. C. Chen, M. Li, Y. Xing, Y. Li, C. C. Joedecke, J. Jin, Z. Yang and D. Liu, *Langmuir*, 2012, **28**, 17743−17748.
